# A realist review protocol on communications for community engagement in maternal and newborn health programmes in low- and middle-income countries

**DOI:** 10.1186/s13643-022-02061-9

**Published:** 2022-09-12

**Authors:** Sara Dada, Aoife De Brún, Esther Namwaba Banda, Sanghita Bhattacharya, Zaccheous Mutunga, Brynne Gilmore

**Affiliations:** 1 UCD Centre for Interdisciplinary Research Education and Innovation in Health Systems (UCD IRIS Centre), School of Nursing Midwifery and Health Systems, University College Dublin, Dublin, Ireland; 2 School of Nursing Midwifery and Health Systems, University College Dublin, Dublin, Ireland; 3 Zambia Ministry of Health, Lusaka, Zambia; 4 Midwives Association of Zambia, Lusaka, Zambia; 5 Public Health Foundation of India, Gurugram, Haryana India; 6 Concern Worldwide, Nairobi, Kenya

**Keywords:** Maternal health, Newborn health, Realist review, Realist synthesis, Community engagement

## Abstract

**Background:**

Community engagement (CE) has been increasingly implemented across health interventions, including for maternal and newborn health (MNH). This may take various forms, from participatory women’s groups and community health committees to public advocacy days. While research suggests a positive influence of CE on MNH outcomes, such as mortality or care-seeking behaviour, there is a need for further evidence on the processes of CE in different settings in order to inform the future development and implementation of CE across programmes. Communication is an integral component of CE serving as a link between the programme and community. The aim of the realist review described in this protocol is to understand *how*, *why*, *to what extent*, and *for whom* CE contributes to intended and unintended outcomes in MNH programming, focusing on the communication components of CE.

**Methods:**

Realist review methodology will be used to provide a causal understanding of what communication for CE interventions in MNH programming work, for whom, to what extent, why, and how. This will be done by developing and refining programme theories on communications for CE in MNH through a systematic review of the literature and engaging key experts for input and feedback. By extrapolating context-mechanism-outcome configurations, this review seeks to understand how certain contexts trigger or inhibit specific mechanisms and what outcomes this interaction generates when communication in CE interventions is used in MNH programming.

**Discussion:**

A realist philosophy is well-suited to address the aims of this study because of the complex nature of CE. The review findings will be used to inform a realist evaluation case study of CE for an MNH programme in order to ascertain transferable findings that can inform and guide engagement activities in various settings. Findings will also be shared with stakeholders and experts involved in the consultative processes of the review (through workshops or policy briefs) in order to ensure the relevance of these findings to policy and practice.

**Systematic review registration:**

PROSPERO CRD42022293564

**Supplementary Information:**

The online version contains supplementary material available at 10.1186/s13643-022-02061-9.

## Introduction

Improving maternal and newborn health (MNH) has been highlighted as a global priority in both the Millennium Development Goals (2000–2015) and the Sustainable Development Goals (2016–2030). MNH refers to “the health of women during pregnancy, childbirth, and the postpartum period . . . and [in] the babies’ first month of life” [1]. Notably, the majority of maternal and newborn deaths occur in low- and middle-income countries (LMICs) [2, 3]. Research on MNH has presented the three delays model which describes delays attributable to the majority of maternal health mortality and outcomes [4]. The delays occur in: (1) the decision to seek care, (2) the identification and access to a health facility, and (3) receiving adequate and appropriate care. The decision to seek care (the first delay) involves both women and their families’ decisions on if and/or when to seek care throughout pregnancy and childbirth. This decision-making process may be influenced by external forces, including community-related factors, representing the importance of addressing MNH at multiple levels — including not only the individual but also the community and overall health systems [4, 5]. Additionally, political, social, and community factors play a key role in accessing health facilities (the second delay) such as through arranging transportation or peer influence on where to seek care [6]. As a result, a number of recommendations and guidelines aim to address the first delay through community-based interventions and activities.

In 2015, the World Health Organization (WHO) released recommendations for community-based health promotion interventions to improve MNH [7]. These interventions, such as women’s groups or mobilizing community volunteers, have been effective approaches to influence maternal and neonatal mortality and/or uptake of care [5, 8, 9]. For example, women’s groups using the participatory learning and action cycle have been found to increase care-seeking during and after pregnancy by involving local women in a process to identify local challenges and prioritize solutions for improving maternal health [10]. Community-based interventions for MNH also promote communication and support, such as community engagement and mobilization strategies [7, 11]. Within this spectrum of interventions, engaging populations has been used to build trust and affect behaviour change through participatory activities and community events in order to deliver care or make community-informed improvements in quality of care [8, 12–16]. These interventions delivered at the community level vary; however, many of them incorporate elements of community engagement (CE).

### Community engagement

Community engagement has become an increasingly employed component of programmes across disciplines because it can empower populations and build capacity for longer-term solutions by promoting participation to improve health [17–20]. While there is a range of definitions of CE, it can broadly be described as “a process of developing relationships that enable stakeholders to work together to address health-related issues and promote well-being to achieve positive health impact and outcomes” [21]. CE is a complex health intervention involving multiple actors and interactions at different levels (e.g. individual, interpersonal, social systems). Terms such as community or social mobilization, participation, community action and empowerment, and communication are often used together and/or interchangeably with CE [19, 22, 23].

The broader outcomes of CE vary with the goals or purpose of the intervention in which it is incorporated [24]. CE has been used to enable community participation, protect communities’ interests, tailor programmes to a community’s needs, build capacity, and encourage programme acceptance [25–27]. Defining the success, effectiveness, or “end game” of CE in the literature has been complex and varied. Richardson et al. reported four potential purposes of CE, including the following: (1) achievement of project goals, (2) change in the community, (3) participant satisfaction, and (4) positive relationships with the community [17]. CE can also have specific ethical goals, described by Dickert and Sugarman as “enhanced protection, enhanced benefits, legitimacy, and shared responsibility” [25].

Researchers and practitioners have explored how CE contributes to achieving these outcomes. Studies on women’s groups, for example, have suggested this intervention increases community capacity to identify MNH problems and mobilize resources to address them [28]. Previous literature has highlighted various factors contributing to effective CE: ensuring two-way dialogues, building and fostering trust, considering and adapting to local contexts and realities, practicing honesty and transparency, and showing respect to community members in both the development and implementation of programmes [13, 22, 27, 29–31]. Frequently adopted components of CE programmes also include mobilizing existing community capacity such as existing groups or structures as well as using local languages and avenues for communication [13, 22, 29, 32]. A framework for CE, developed from observations of communities during the Ebola vaccine trials in Sierra Leone, describes four principles present in the observed CE programme: reciprocity, relatability, relationships, and respect [13]. Evidence suggests that these four principles may be causally linked to the trial’s recruitment and participation outcomes and have also been identified across the literature in a range of settings and interventions such as in infectious disease research, vaccination trials, and health facility committees [13, 27, 31, 33–37].

In addition to these factors, local contexts and histories, social structures and power dynamics, and community and individual beliefs are essential elements to consider in CE programmes [13, 37, 38]. For example, in the West Africa Ebola outbreak, it is impossible to ignore how socio-political and historical factors influenced the perception of both local and foreign government intervention during the crisis [32]. The recent civil wars and the challenging process of rebuilding trustworthy structures of governance could explain, in part, the community resistance and mistrust that was common throughout the epidemic; this was also reflected in the approaches of CE during the response [13, 32, 39–41]. Existing relationships and social hierarchies, socioeconomic status, cultural beliefs and values, the state of the health system, and community perceptions and knowledge are all contextual factors to be considered in the development and implementation of CE strategies [13, 22, 24, 32, 35, 37, 42, 43].

A number of factors have been described above as important considerations in the development or application of CE, yet the literature is limited when it comes to how and why these factors are important and/or influential, especially across different contexts. This may be in part because the research in this field is often focused on reporting health outcomes without a clear understanding of what causes these outcomes [19]. As a result, it is challenging to extrapolate which components or processes of CE may be transferrable across different health programmes. Developing a better causal understanding of how and why CE programmes work, or do not, will have implications for policy and practice by informing the translation of findings into recommendations for integrating and scaling up these CE interventions for MNH.

### Realist approach

While the wide and varied literature on CE describes its associations with specific outcomes (e.g. mortality rate) and many interventions claim to incorporate elements of CE, uncertainty remains around what principles and practices make CE “effective” across varied contexts [13, 29, 44]. A realist approach can be used to examine this. Realist philosophy is based on the ontological assumptions that there is a “real world” which includes open social systems and structures with components that interact and have consequences [45, 46]. Notably, realist philosophy emphasizes that programmes can work differently for different people in different circumstances. This can be applied to CE by investigating a programme’s generative causation — essentially which mechanisms (M) underpin effective CE interventions and the contexts (C) that trigger these mechanisms to generate the observed outcomes (O). The complexity of CE programmes as well as the importance of context in implementing CE make this subject well-suited for a realist synthesis [17, 24, 47]. A realist review can be used to provide a deeper understanding of what CE interventions in MNH work, for whom, to what extent, why, and how [48]. Realist approaches do this by using context-mechanism-outcome configurations (CMOCs) to describe how generative causation within a system works and which mechanisms are triggered in specific contexts and how this interaction leads to intended or unintended outcomes [49, 50].

As described previously, the local contexts and settings in which CE interventions are implemented have been recognized as influential components to the programmes. A realist approach is useful to explicitly understand and document this. Furthermore, the importance of generative causation that underpins the realist philosophy acknowledges that social systems influence individuals and vice versa [47]. This affects our conceptualization of CE as more than one-sided implementation but as a dynamic process that may also change the context in a setting [51]. The realist approach acknowledges that complex interventions succeed in some situations and for some individuals but not in others because of differences in processes, participants, and contexts [52]. The purpose of a realist review is to be explanatory and provide insight on how and why these different outcomes are generated in different contexts [48, 53]. This understanding of how CE programmes work in specific contexts to trigger the mechanisms that lead to observed outcomes can be useful to inform the design of programmes and policies [53].

### Aim

The realist review described in this protocol will aim to understand *how*, *why*, *to what extent*, and *for whom* communications in CE contribute to intended and unintended outcomes in MNH programming. This will be done by developing and refining initial programme theories (IPTs) on communications for CE in MNH through reviewing the literature and engaging an expert advisory committee for input and feedback. In order to limit the scope of the review to circumstances specific to this type of programming, this review will focus on MNH interventions and programmes. However, consistent with the realist approach, the scope and specific question for this review have been iteratively refined and further narrowed to specifically consider the communications component of CE [54, 55]. While this review will be conducted and reported according to the Realist and Meta-narrative Evidence Syntheses: Evolving Standards (RAMESES) publication standards for realist syntheses [56], reviewers have also populated the PRISMA-P checklist to provide additional oversight in the methodology of this review (Additional file 1). This realist review protocol is registered with PROSPERO (CRD42022293564).

## Methods

### Phase 1, step 1: Define review scope and form expert advisory committee

**Fig. 1:**
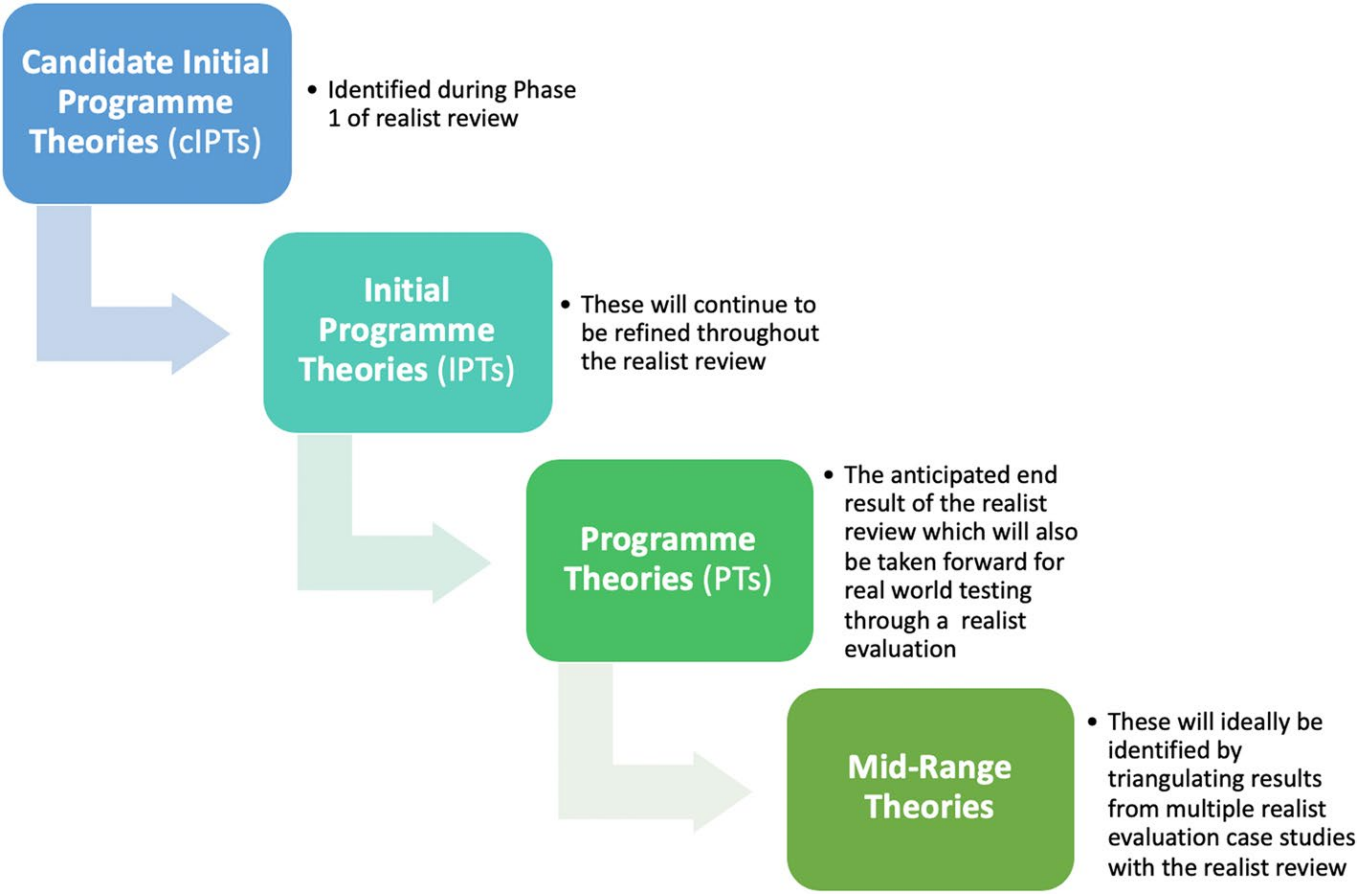
The process of theory development and refinement to be followed in the realist review process

Realist reviews are iterative by nature, and the refinement of theory happens throughout the review and informs the review itself. Figure 1 maps the progression of theory development and refinement throughout the realist process. This figure can be referred to in order to understand the different “versions” or stages of the theories.

**Fig. 2:**
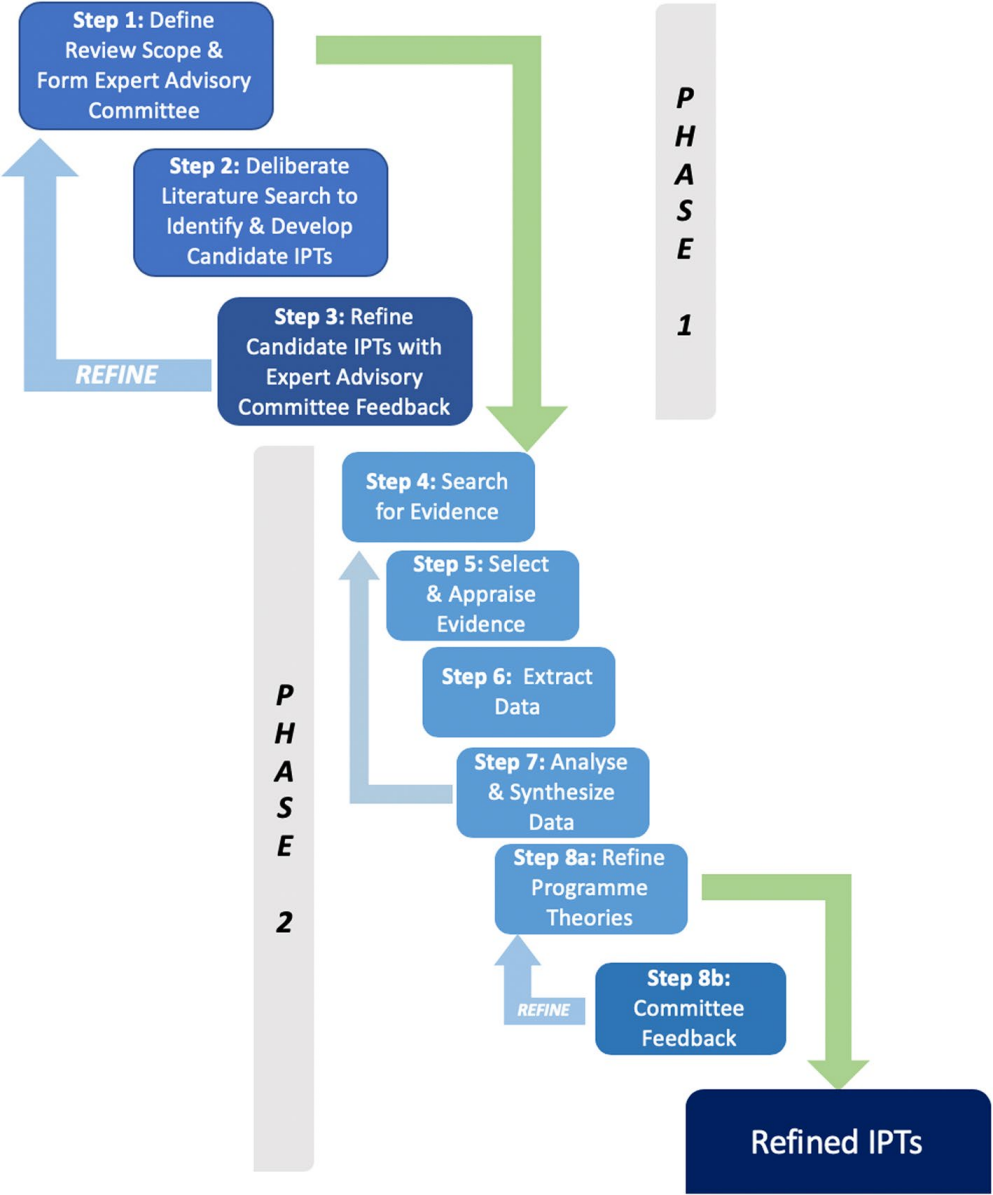
Realist review steps demonstrating the iterative nature of theory refinement throughout the review process

Additionally, the process and the overall steps of the realist review laid out by this protocol are exhibited in Fig. 2. This process is based on the methodology described by Pawson et al. [48], and the figure is adapted from Power et al. [57]. Phase 1 and phase 2 together represent the entirety of the realist review. Phase 1, the development of this realist review protocol, has been completed and is presented in this paper. This phase, as depicted in Fig. 2, describes the process that was used to design the realist review and develop the candidate IPTs that will be refined during phase 2, which includes the systematic search and data extraction and synthesis.

A relevant scoping review is ongoing with the aim of establishing the definitions and descriptions of CE in the literature, which will further inform this realist review [23]. Given there is a wide range of terminology and definitions used in the CE literature, this initial scoping review will collate these definitions and key terms as well as clarify the conceptualization of CE [58]. This process has also been used as a broad, preliminary search to familiarize reviewers with the literature as well as reveal some of the key issues and frameworks used in CE interventions.

Aligned to realist review methodology, a committee of key experts will be consulted throughout the realist review process [24, 44, 53, 56, 59]. This six-person advisory committee is comprised of representatives from the World Health Organization and academia as well as in-country partners and practitioners with experience working with CE and/or MNH. The role of this advisory committee is to provide feedback on the protocol and findings which has already been used to inform phase 1 of this review identifying key literature, incorporating relevant reflections and experiences, and providing feedback on the candidate IPTs. The initial questions posed to the expert advisory committee can be found in Additional file 2. This is included to provide further explanation of how this group of experts was engaged in this process. In phase 2, this group will continue to be consulted as they provide input on the theory refinement process.

### Phase 1, step 2: Deliberate literature search to identify and develop candidate IPTs

A deliberate literature search was conducted to develop candidate IPTs. In order to be useful from a realist perspective, this initial search and candidate IPT development also considers potential contexts, mechanisms, and outcomes of CE. This process has involved reviewing known, fundamental studies and drawing on reflections from the lead researcher’s experiences. For example, the previously mentioned framework for CE developed in Sierra Leone served as a starting point in the consideration and development of candidate IPTs [13]. The deliberate literature search involved soliciting the expert advisory committee for recommended documents and reviewing those with sufficient descriptions of the CE programme that could then be used to develop IPTs. The expert advisory committee also provided general comments on their experiences and knowledge of CE that were used to further inform the search for background literature.

### Phase 1, step 3: Refinement of candidate IPTs with expert advisory committee feedback

Members of the expert advisory committee received a draft of five candidate IPTs to review and provide feedback (Additional file 3). The process of reviewing this feedback informed further narrowing the scope of this review to focus on communication for community engagement because this aspect was present across the range of candidate IPTs and emphasized by various experts. Communication has been integral across interventions for behaviour change, including public health and development initiatives, and plays a significant role in CE programmes, for example through providing information or building trust [60–62]. Health communication has been defined as “methods to inform and influence individual and community decisions that enhance health” [63]. CE communications for the purpose of this realist review refers to the approaches, strategies, and messaging content or channels used to communicate with the community in or for the CE programme. Notably, literature on communication has also pointed to the use of context-specific theories to explain communications [64].

Previous literature on realist programme theory suggests using pre-existing theories or frameworks to inform and organize IPT development [65]. Based on this, the candidate IPTs were refined into three IPTs reflecting the first three phases of the communication cycle described by the communication for development (C4D) tool: initial planning/setting identification, strategy design, and implementation [66]. A number of theories and approaches to health communications have begun to explain the role of communications in health promotion or behaviour change campaigns. For example, substantive theories, like the theory of interpersonal relationships, point to the importance of the relationship between the individual/patient and the health worker/implementer as a source of motivation to engage with a programme because of the empathy and trust built in this relationship [67, 68]. Two other common theories centred on individuals’ cognitive logic are the health belief model and the theory of planned behaviour (previously known as the theory of reasoned action) [19, 69, 70]. Both of these theories describe how individuals’ beliefs or understanding of something influence their behaviour — and these beliefs can be informed through communication [69]. The theory of planned behaviour proposes how beliefs about attitudes, norms, and control influence intention and therefore behaviour [19, 70, 71]. This applies to communications by highlighting how approaches that not only inform and educate community members but also incorporate local design and collaboration can influence behavioural beliefs by being contextually relevant and appropriate [19].

**Fig. 3:**
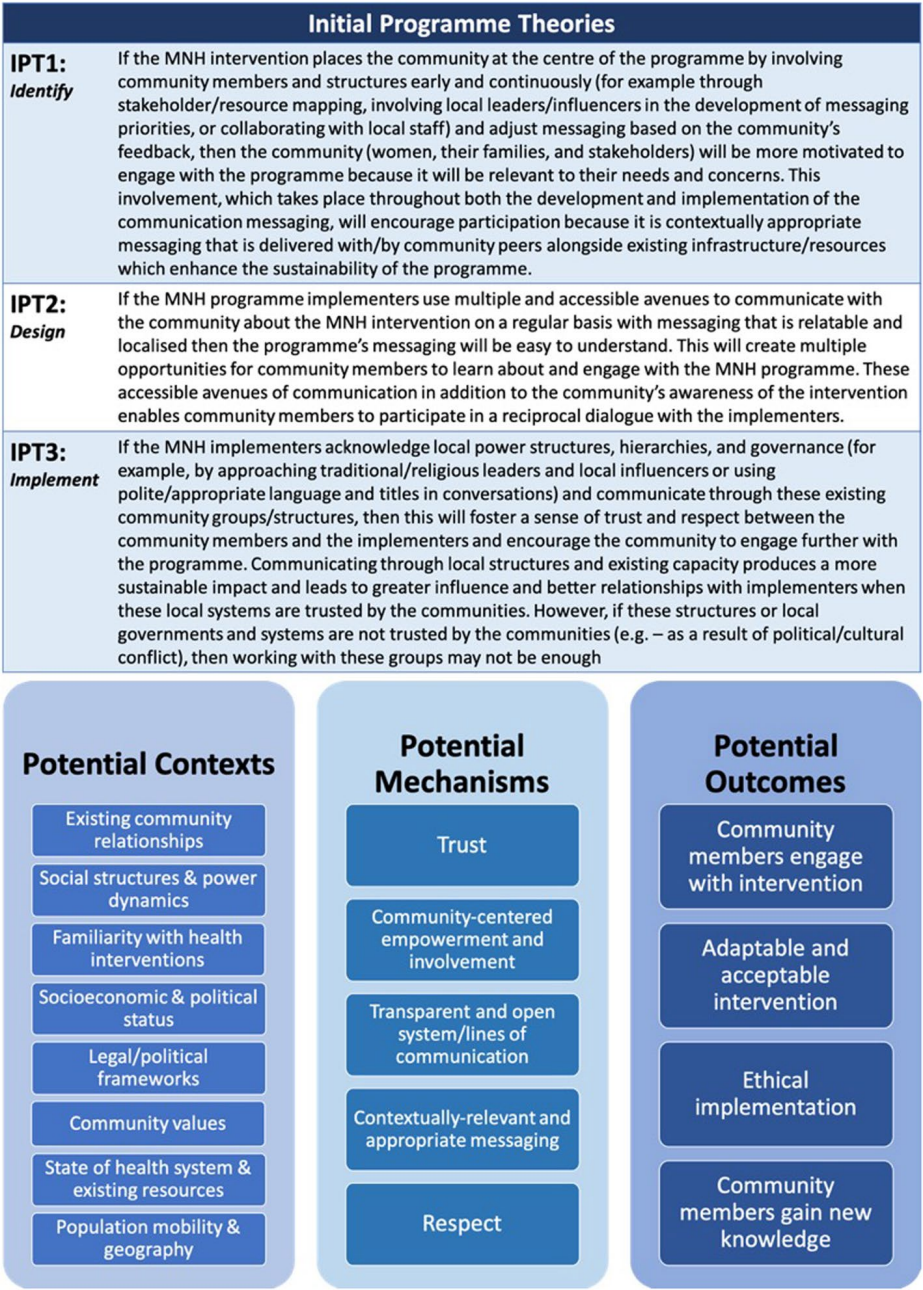
Initial programme theories and potential contexts, mechanisms, and outcomes for communications in community engagement in maternal and newborn health as identified during phase 1 of the realist review. The contexts, mechanisms, and outcomes are currently not configured and are not an exhaustive list, as this will be informed by the review process

Figure 3 provides the resulting IPTs after incorporating feedback from the committee as well as additional research on communications theories described above. This figure represents the findings from phase 1 of this review which will move forward to be refined during phase 2. Consistent with other realist reviews, this figure also includes lists of potential contexts, mechanisms, and outcomes but does not present configured CMOs [24, 57]. The CMOCs will be informed by and developed through the data extraction and synthesis process.

### Phase 2, step 4: Search for evidence

The first step in phase 2 involves searching for evidence in the literature based on the elicited candidate IPTs from phase 1. The terms used for this search strategy have been informed by key publications in the relevant fields. Table 1 includes the initial search terms with the major concepts of MNH and CE terms. These specific terms were used in order to capture documents with activities that are explicitly named as CE-related programmes. The search uses the Boolean operator “OR” in between terms as noted below and “AND” in between concepts. This broad search strategy was last conducted on 01 October, 2021, across seven databases (PubMed/MEDLINE, Embase, CINAHL, PsycINFO, Scopus, Web of Science, Global Health) for a scoping review on CE and MNH programming [23]. OAIster and OpenGrey were also searched using keywords for grey literature with relevant insight. The resulting records provide a useful starting point to review a large number of articles for potential relevancy.

**Table 1:** Search terms

Problem: **MNH terms** (title/abstract)	Antenatal OR prenatal OR pregnan* OR matern* OR “child health” OR “newborn health” OR postpartum OR postnatal OR perinatal OR reproductive OR birth OR “family plan*” OR neonat* OR ANC OR PNC OR MNCH OR RMNCH
Intervention: **community engagement terms** (all fields)	“Citizen participation” OR “citizen engagement” OR “collaborative partnership” OR “community action” OR “community advisory” OR “community consultation” OR “community collaboration” OR “community engagement” OR “community involvement” OR “community mobilization” OR “community mobilisation” OR “community liaison” OR “community network*” OR “community participation” OR “grassroots participation” OR “grassroots network*” OR “public engagement” OR “public participation” OR “public representation” OR “participatory action” OR “participatory learning” OR “stakeholder engagement” OR “social engagement” OR “social accountability”
Time	Limit 2000 — current

The bibliographies and reference section of all relevant articles will also be hand searched for potential additional inclusion. CLUSTER searching may also be a useful search technique in this process [72]. This will involve reviewers hand-searching citations of key studies and contacting lead authors for additional recommendations and/or unpublished materials that share a common thread and then searching Google Scholar for relating authors or sources providing insight on specific programme theories [54, 72]. These search methods are useful in a realist review because they provide a systematic approach to reviewing related studies that can provide different angles of insight. The search process in a realist review is iterative, and any subsequent alterations in focus, search terms, or strategy will be documented as they occur [53, 54, 56, 73].

The timeframe is limited to 2000 in order to reflect the increase in attention and literature on CE over the last two decades and to capture the most relevant evidence. While the search does not include a geographic limitation, the relevance of studies based on their setting will be considered in the next step of selecting and appraising evidence. The IPTs described in Fig. 3 will be used to inform the screening process. In line with the realist approach, this search will be iterative, and any changes to the search strategy, such as incorporating more specific terms relating to components of theory, will be documented accordingly [74].

### Phase 2, step 5: Select and appraise evidence

Due to the iterative nature of searching for evidence in a realist review, the search for empirical evidence continues to be informed as documents are identified [54, 75]. Additionally, though no study will be excluded based on the language, the searching process will be conducted in English. The results from the database searches will be uploaded into Covidence, an online review software, where they will be screened for inclusion. These records as well as additional references that are identified throughout the iterative search process will also be collected and managed in an EndNote library.

**Fig. 4:**
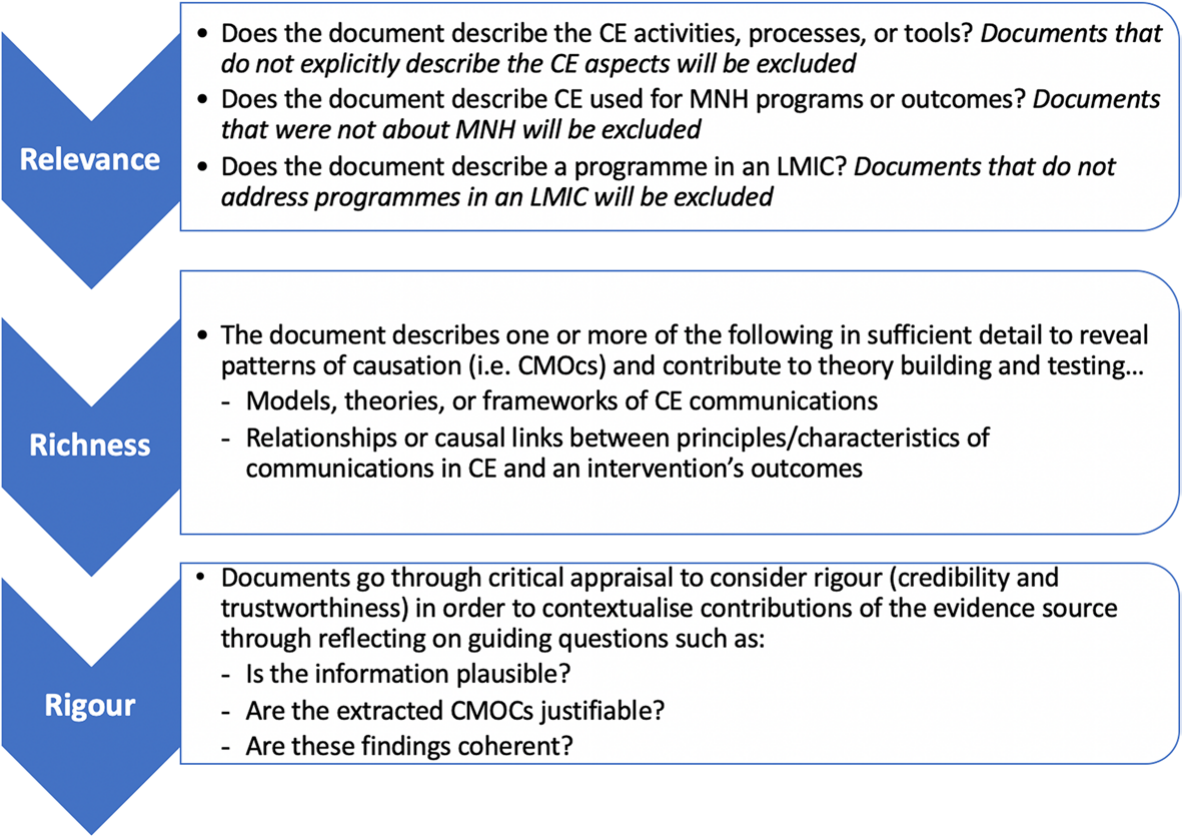
Full text inclusion criteria for relevance as well as processes to assess richness and rigour

Documents will be reviewed based on their relevance, richness, and rigour (Fig. 4), in line with the RAMESES guidelines [53, 56]. While two reviewers will screen documents for initial relevance (with discrepancies adjudicated by a third reviewer), one reviewer will further assess the included documents for richness and rigour. A second reviewer will review these appraisals as a validation check, and any discrepancies or questions will be discussed amongst the reviewers. In line with standard practice for realist reviews, this realist review will not exclude any evidence based on rigour; rather, publications with insufficient information to contribute to programme theory will be excluded [53, 57]. Any documents providing relevant evidence will be included in this review, and the results of their critical appraisal will be included for transparency. Reviewers will seek out multiple sources of data for any aspect of programme theory as well as seek to build coherent and credible arguments for programme theory [55].

### Phase 2, step 6: Extract data

A data extraction form has been developed based on the cIPTs and the aims of the review (Table 2). This will be reviewed and refined throughout the review process as necessary. The following data will be extracted from the studies and cases identified for inclusion: setting and objectives of programme, CE activities, any models or theoretical frameworks that informed the CE, and the CMOCs of the CE. Data providing evidence to confirm, refute, or refine the IPTs will be documented as well as notes on this decision-making process and any potential knock-on effects or linkages to other IPTs/relevant findings. Two reviewers will independently extract an initial sample of documents and compare. A small sample of data extraction forms will also be shared with the advisory committee for feedback and refinement.

**Table 2:** Data extraction form

**Document details**				
Author	Year	Publication type	Aim/objectives	Setting
Target population	Study design/methods	Findings	Description of CE activities	Models/theoretical frameworks
***Contexts, mechanisms, outcomes, and theory refinement***				
**CMOC 1**	Context	Mechanism	Outcome	Additional information
***Relevant IPT(s)***	Confirm/refute/refine	Suggested revisions	Decision-making process	Linkages/knock-on effects
**CMOC 2**	Context	Mechanism	Outcome	Additional information
***Relevant IPT(s)***	Confirm/refute/refine	Suggested revisions	Decision-making process	Linkages/knock-on effects
**CMOC 3**	Context	Mechanism	Outcome	Additional information
***Relevant IPT(s)***	Confirm/refute/refine	Suggested revisions	Decision-making process	Linkages/knock-on effects

### Phase 2, step 7: Analyse and synthesize data

The CMOCs identified in the included documents will be analysed. This data will be synthesized by reviewing how the CMOCs contribute to the IPTs for theory refinement. Realist approaches use abductive and retroductive reasoning to synthesize data and draw conclusions [76]. Abduction uses the evidence at hand to make a logical inference or conclusion. Retroduction is about uncovering causal forces. This may be informed by inductive and deductive reasoning or individual insight on generative causation that may come from the ability to identify patterns or changes in those patterns [76–78].

This review will follow the approach to data analysis and synthesis outlined by Gilmore et al. [78]. Data extraction forms will be uploaded into NVivo12 (QSR International Pty Ltd.) and coded according to a codebook informed by the IPTs and data extraction [78]. In this process, each IPT will be a “node” in the NVivo workbook, and the extracted CMOCs will be linked to the appropriate IPT node with a note describing how the data calls for supporting, refuting, or refining the theory. This codebook may be amended during the data synthesis process as new codes and patterns emerge.

### Phase 2, step 8: Refine programme theory and incorporate expert advisory committee feedback

In the process of refining initial programme theories, an additional search may be conducted to provide evidence and literature on wider substantive theory that can aid in explanation and refinement of the theory [53–55]. At this point in the review, theories may be added, refuted, and refined. The refined initial programme theories and their respective evidence will be presented to the expert advisory committee who will provide feedback on the programme theory, contributing to the iterative refinement that is characteristic of a realist review. Additionally, it may be useful to consider bringing in additional local stakeholders with practical knowledge and expertise to reflect on and workshop the initial programme theories and inform findings from the review to be most useful in the field [44].

## Discussion

The purpose of this realist review will be to refine and develop IPTs that explain “how, why, to what extent, and for whom” communication for CE in MNH works within LMICs. By identifying contextual factors, the mechanisms they trigger, and the intended and unintended outcomes of CE, this realist review will provide an understanding of the generative causation of CE in these settings. These findings will address an identified gap in the literature to move beyond the binary outcomes of CE to answer the questions of what is causing these outcomes [19]. A previous realist review on CE with health research highlighted the importance of developing “working relationships” between communities and researchers and the contextual factors and mechanisms that influence the CE and research [43]. However, this previously published review focuses on the research setting which has different implications including elements of study design, processes of informed consent, and other ethical considerations which would influence the patterns of generative causation that explain how, why, to what extent, and for whom CE works.

The findings of this realist review will have not only theoretical impact but also practical impact by informing the future planning and design of CE communications for MNH interventions. The IPTs refined throughout the review process can be further studied for their potential transferability across settings and programmes and serve as an evaluation starting point for researchers investigating CE in other fields. The ultimate goal of this research is to develop a better understanding of CE in MNH that can be translated to recommendations for policy and practice.

### Strengths and limitations

There are several strengths of a realist approach that make it well-suited to consider CE for MNH. CE is a complex process itself, and realist philosophy acknowledges and addresses the dynamic nature of these interventions [79]. Notably, context is often emphasised as an integral consideration in developing CE interventions [13]. An intervention in one setting may see a range of different responses from the audience it targets because of their unique experiences and perceptions. By examining *both* the contexts and mechanisms that lead to intended or unintended outcomes, realist approaches are applicable to study the variations in where and how CE work is conducted [50]. Additionally, there are many different forms and examples of community engagement activities. A realist approach can be used to unpack the generative causation in these processes by determining which contexts enable or inhibit specific mechanisms that lead to outcomes across various forms of CE.

There are a number of limitations to consider and be aware of in executing this realist review. While substantive theory on CE will be incorporated, this review will focus on literature relating to CE in MNH programmes specifically. However, it is recognized that CE used in other types of programmes could provide important insights. A potential challenge of this review will be the large variation in CE literature as well as the range of different definitions and terminology used to refer to engagement versus involvement, participation, mobilization, etc. Reviewers will attempt to account for some of these limitations by documenting decisions and review processes to ensure transparency and potential replicability [55, 56]. Finally, it may be challenging to involve programme end users on the expert advisory committee who could provide an alternative perspective from the academic and policy lenses.

### Dissemination and next steps

This protocol will be used to guide and conduct the described realist review. The results of this review will be written up according to the RAMESES publication standards for realist syntheses and submitted for publication in a peer-reviewed journal [56]. Additionally, to encourage dissemination and practical application beyond the sphere of academia, findings will be shared with the stakeholders and experts involved in the consultative processes of the review (for example through an interactive workshop and preparations of policy briefs). Finally, these review findings and IPTs will be used to inform the next stage of this project: a realist evaluation of CE for an MNH programme. This realist evaluation will enable further refinement of the programme theories through real-world investigation. The goal of this body of work is to better characterize and understand CE in order to present an adaptable CE framework (in the form of CMOCs and/or programme theories) that can inform and guide engagement activities in various settings.

## Supplementary Information

**Additional file 1.** PRISMA-P 2015 Checklist

**Additional file 2.** Initial Queries to Expert Advisory Committee

**Additional file 3.** Candidate Initial Programme Theories Shared with Expert Advisory Committee for Feedback

## Data Availability

Data sharing is not applicable to this article as no datasets were generated or analysed.

## Abbreviations

CE: Community engagement
MNH: Maternal and newborn health
LMIC: Low- and middle-income countries
WHO: World Health Organization
CMOC: Context-mechanism-outcome configuration
RAMESES: Realist and Meta-narrative Evidence Syntheses: Evolving Standards
cIPT: Candidate initial programme theory
IPT: Initial programme theory
PT: Programme theory
C4D: Communication for development
