# Supplementary material for: A realist review protocol on communications for community engagement in maternal and newborn health programmes in low- and middle-income countries
Source: Syst Rev. 2022 Sep 12;11:201. doi: 10.1186/s13643-022-02061-9 (PMC9465973; doi:10.1186/s13643-022-02061-9)
Supplement: Supplementary file 2 — Additional file 2. Initial Queries to Expert Advisory Committee [file 13643_2022_2061_MOESM2_ESM.docx]

# Additional File 2: Initial Queries to Expert Advisory Committee

As a reminder I am conducting this realist review to develop some initial programme theories on how/for whom/to what extent community engagement is effective in MNH. Your answers to these questions will be used to conduct the background search to inform the first formulations of those candidate theories.

1. Are there any **seminal papers or literature** that you would recommend that describe how community engagement programs have worked?
2. What do you believe are some of the **key theories behind how community engagement works**?
3. What do you think are the **top three “active ingredients”** or must haves for an effective community engagement program?
4. What are the **top three challenges or problematic issues**faced by community engagement programs?
5. Are there any **unique considerations** for community engagement work operating in **LMICs**?

Please let me know **if you prefer** to have a quick chat about your thoughts on these questions rather than responding via email.
