# Supplementary material for: A realist review protocol on communications for community engagement in maternal and newborn health programmes in low- and middle-income countries
Source: Syst Rev. 2022 Sep 12;11:201. doi: 10.1186/s13643-022-02061-9 (PMC9465973; doi:10.1186/s13643-022-02061-9)
Supplement: Supplementary file 3 — Additional file 3. Candidate Initial Programme Theories Shared with Expert Advisory Committee for Feedback [file 13643_2022_2061_MOESM3_ESM.docx]

# Additional File 3: Candidate Initial Programme Theories Shared with Expert Advisory Committee for Feedback

*Instructions: Please provide any comments/suggestions you have on the following theories. This may include (but is not limited to) which aspects are important or missing or need further clarification. Please feel free to email me at* [*sara.dada@ucdconnect.ie*](mailto:sara.dada@ucdconnect.ie) *if you have any further questions or if you would prefer to schedule a short chat to provide any feedback verbally.*

**cIPT1:** If the MNH programme implementers use multiple and accessible avenues to communicate with the community (print, radio, group meetings, etc.) about the MNH intervention on a regular basis with messaging that is relatable, then the programme’s messaging will be easy to understand. This will create multiple opportunities for community members to learn about and engage with the MNH programme. These approachable and accessible avenues of communication in addition to the community’s awareness of the intervention enables community members to participate in a reciprocal dialogue with the implementers.

**cIPT2:** If the MNH implementers listen and respond to community needs and feedback by modifying the intervention (throughout its implementation) and are transparent about how the community’s feedback has influenced the intervention, then the community (both women and their families) will be more motivated to engage and participate in the intervention because it will be relevant to their needs and concerns and they will feel respected by the implementers because their concerns have been heard and responded to.

**cIPT3:** If the MNH intervention places the community at the center of the program by involving community members early and continuously (for example through with local stakeholders on design and recruiting/collaborating with local staff for implementation), then the programme will be appropriate to the local setting’s needs/contexts and will encourage women and their families to participate because the intervention is being delivered with/by their community peers.

**cIPT4:** If the MNH implementers acknowledge local power structures, hierarchies, and governance (for example, by approaching local traditional or religious leaders or using polite/appropriate language and titles in conversations), then this will foster a sense of trust and respect between the community members and the implementers and encourage the community to engage further with the programme.

**cIPT5:** If the MNH implementers involve existing programmes and community groups/structures within and around the health system, then the community will be more likely to participate in the MNH intervention because these groups have already built trust within the community and have the opportunity to produce more sustainable impact by building off of their existing capacity. However, if these structures or local governments and systems are not trusted by the communities (e.g. - a result of political/cultural conflict), then working with these groups may not be enough.
